# Radiated tumor cell-derived microparticles effectively kill stem-like tumor cells by increasing reactive oxygen species

**DOI:** 10.3389/fbioe.2023.1156951

**Published:** 2023-06-05

**Authors:** Yan Hu, Chao Wan, Xiao Yang, Yu Tian, Suke Deng, Dandan An, Yijun Wang, Jiacheng Wang, Zhiyun Liao, Jingshu Meng, You Qin, Yajie Sun, Kunyu Yang

**Affiliations:** Cancer Center, Union Hospital, Tongji Medical College, Huazhong University of Science and Technology, Wuhan, China

**Keywords:** radiated tumor cells-derived microparticles, stem-like tumor cells, chemotherapy resistance, quiescence, reactive oxygen species

## Abstract

Stem-like tumor cells (SLTCs) are thought to be the cellular entity responsible for clinical recurrence and subsequent metastasis. Inhibiting or killing SLTCs can effectively reduce recurrence and metastasis, yet little has been done to clear SLTCs because they are usually resistant to chemotherapy, radiotherapy, and even immunotherapy. In this study, we established SLTCs by low-serum culture and confirmed that the low-serum-cultured tumor cells were in a quiescent state and resistant to chemotherapy, showing features of SLTCs, consistent with the reported data. We demonstrated that SLTCs had high levels of reactive oxygen species (ROS). Based on the finding that radiated tumor cell-derived microparticles (RT-MPs) contained ROS, we used RT-MPs to kill SLTCs. We found that RT-MPs could further increase ROS levels and kill SLTCs *in vivo* and *in vitro* partially by ROS carried by the RT-MPs themselves, providing a new method for eliminating SLTCs.

## 1 Introduction

Recurrence and metastasis pose significant challenges to tumor treatment. Both recurrence and metastasis can be caused by residual tumor cells that remain in the body after initial treatment, which may enter into a dormant state and are resistant to conventional therapies such as chemotherapy and radiotherapy (Saleh et al., 2019; Summers et al., 2020). Notably, the development of donor-derived metastasis was found in patients who have received organ transplantation from individuals who had been cured of melanoma or glioblastoma, suggesting that some latent tumor cells can escape immune surveillance and grow in an immunosuppressed environment, leading to the development of metastatic disease (MacKie et al., 2003; Xiao et al., 2013). It is thought that dormant tumor cells, also known as stem-like tumor cells (SLTCs), are the cellular entity responsible for clinical recurrence and subsequent metastasis. SLTCs are present in primary tumors as a rare subpopulation and their percentage correlates with metastatic potential (Lawson et al., 2015; Zhang et al., 2017). SLTCs have considerable tumor-initiating capacity, and can proliferate and differentiate to produce advanced metastatic disease (Lawson et al., 2015; Nallasamy et al., 2022). However, their nature and how to effectively kill or inhibit them remain largely unknown.

Cellular dormancy is regulated by various signals derived from endothelial cells and immune cells (Widner et al., 2018; Endo and Inoue, 2019). TGF-β and IFN-β have recently been identified as important inducers of cell dormancy (Lan et al., 2019; Miao et al., 2019). Joan and his group isolated dormant cancer cells from early-stage human lung and breast carcinoma cells (Malladi et al., 2016). They found that dormant cells showed stem cell-like characteristics and expressed SOX2 and SOX9 transcription factors. They demonstrated that mitogen-low media (MLM, 2% serum) could effectively support the recapitulation of critical features of slow-cycling, long-lived SLTCs.

Oxidative stress exerted by reactive oxygen species (ROS) is generally detrimental to cells, and the redox status of tumor cells usually differs from that of normal cells (Hayes et al., 2020). Due to their high metabolic rate and activation of oncogenic pathways, tumor cells exhibit elevated ROS levels (Gorrini et al., 2013; Kudo et al., 2020). This makes them more susceptible to the harmful effects of ROS and provides a therapeutic opportunity for cancer treatment. ROS are also involved in the renewal and differentiation of normal stem cell (Khacho et al., 2016). Although SLTCs share similar phenotypes with normal stem cells, relatively little is known about their redox status.

Extracellular vesicles have emerged as major mediators for intercellular communication and represent a heterogeneous group of cell-derived membranous spherical structures, comprising apoptotic bodies, microparticles (MPs) and exosomes (van Niel et al., 2018; Marar et al., 2021). In our previous studies, we found that radiated tumor cell-released microparticles (RT-MPs) could effectively kill tumor cells and mediated the radiation-induced bystander effect. Both *in vitro* and *in vivo*, RT-MPs exhibited broad killing effects on various types of tumors, and significantly inhibited tumor growth (Wan et al., 2020). In addition to their direct cytotoxic effects, RT-MPs also reprogrammed tumor-associated macrophages to the M1-like proinflammatory phenotype, while the hypoxia and Ultraviolet radiation-treated tumor cell-derived MPs have been reported to polarize M2 phenotype for tumor progression (Ma et al., 2016; Chen et al., 2020; Wan et al., 2020; Zhai et al., 2022). Since SLTCs are resistant to traditional chemotherapy, finding a way to kill these quiescent tumor cells is crucial for the inhibition of tumor recurrence and metastasis (Recasens and Munoz, 2019; Talukdar et al., 2019). In this study, we confirmed that low mitogen and serum culture *in vitro* could promote tumor cells in a quiescent state and resistance to chemotherapy, showing features of SLTCs, and SLTCs had high levels of ROS. RT-MPs could also effectively kill SLTCs by further increasing ROS levels.

## 2 Materials and methods

Chemical reagents. L-glutathione (G4251) was purchased from Sigma-Aldrich (Darmstadt, Germany). H2DCFDA (D399) was bought from Invitrogen (Massachusetts, The United States).

### 2.1 Cell lines and cell culture

Cell lines, including the human lung carcinoma cell lines A549 and murine Lewis, were bought from the American Tissue Culture Collection (ATCC). Luciferase-stably-transfected cell lines (LLC-LUC) were established for *in vivo* studies. Cells were cultured in complete medium (supplemented with 10% fetal bovine serum (FBS), 100 U/mL penicillin, and 100 U/mL streptomycin) at 37°C with 5% CO_2_, and SLTCs were cultured using 2% serum medium. *Mycoplasma* infection was detected routinely and confirmed to be negative.

### 2.2 Isolation of RT-MPs

5 × 10^6^ tumor cells, plated into 10-cm cell culture dishes, were irradiated with a single dose of 20 Gy by 6 MV X-rays (600 MU/min; Trilogy^®^ System Linear Accelerator, Varian Medical Systems), followed by a replacement of 20 mL complete medium. And 72 h later, the collected medium was centrifuged at 1 000 g for 10 min and then 14,000 g for 2 min at 4°C in order to remove tumor cells and debris. After that, the supernatant was centrifuged at 14,000 g for 1 h for the isolation of RT-MPs. The precipitate was washed twice with sterile 1× phosphate buffer saline (PBS) and resuspended in sterile 1 × PBS for *in vivo* experiments or resuspended in complete medium or 2% serum medium for cell experiments.

### 2.3 Transmission electron microscopy

In order to observe their size and morphology, RT-MPs were stained with 2% phosphotungstic acid solution for 5 min and then placed on a copper mesh for TEM imaging (HT7700-SS/FEI Tecnai G20 TWIN, Massachusetts, The United States).

### 2.4 Cell viability study

In order to measure cell viability, 5 × 10^3^ tumor cells per well, seeded into 96-well plates (NEST, Wuxi, China), were treated with different doses of RT-MPs or DDP for 48 h. After that, cell viability was detected by a CCK-8 assay kit (BS350B, Biosharp, Hefei, China).

### 2.5 Colony formation assay

For the purpose of testing the inhibitory ability of RT-MPs towards tumor cells *in vitro*, tumor cells, plated in 48-well plates (NEST; 1 × 10^4^ cells per well), were treated with various concentrations of RT-MPs for 3 days. After that, the cells were fixed with 4% formaldehyde for 30 min, stained with 2% crystal violet (#HT90132, Sigma-Aldrich) for 12 h, and washed with water for three times. The stained cells were finally photographed.

### 2.6 Analysis of ROS production

Tumor cells, plated in 12-well dishes (NEST; 5 × 10^4^ cells per well), were treated with RT-MPs for 4, 12, and 24 h. Subsequently, the cells were stained with H2DCFDA (10 μM) in 1 mL medium without FBS for 30 min at 37°C. After 3 times washes with 1× PBS, the cells were collected, resuspended in 150 μL 1× PBS and detected by flow cytometry (Beckman CytoFLEXS, California, The United States). A minimum of 10,000 cells were acquired from each sample for analysis.

### 2.7 EdU proliferation Assay

A549 and Lewis cells were seeded in 96-well plates, in which 5×10^3^ cells per well were cultured with normal medium and 1×10^4^ cells per well were cultured with 2% serum medium for 72 h, respectively. Cell proliferation was determined with the EdU Apollo *in Vitro* Imaging Kit (RIBOBIO, Guangzhou, China) according to the manufacturer’s protocol.

### 2.8 CFSE proliferation Assay

A549 and Lewis cells were stained with 10 µM CFSE for 10 min and then seeded in 12-well plates (NEST; 1×10^5^ cells per well) with normal medium or 2% serum medium, respectively. After 72 h, the cells were harvested and detected by flow cytometry.

### 2.9 *In vitro* cellular uptake assay

To detect the uptake of RT-MPs, A549 and Lewis, stem-like A549, and stem-like Lewis cells were seeded in a glass-bottom cell culture dish (NEST; 1 × 10^5^ cells per well) and incubated with PKH-26-labelled RT-MPs for 2 h and 4 h. After that, the cells were washed 3 times with 1× PBS and then stained with 10 μM CFSE for 10 min. Subsequently, they were washed 3 times with 1× PBS, fixed with 4% paraformaldehyde for 30 min, and washed again with PBS. The cells were imaged through confocal laser scanning microscopy (A1R/A1, Nikon, Tokyo, Japan). In order to quantify cellular uptake, the cells, seeded in 6-well cell culture dishes (NEST; 1 × 10^6^ cells per well), were treated as described above, washed with 1× PBS for 3 times, collected, and resuspended in 1× PBS (150 μL) for flow cytometry analysis.

### 2.10 RT-qPCR

Total RNA of tumor cells was isolated by the Total RNA Kit IR6834 (Omega Bio-Tek, Georgia, The United States) and measured through NanoDrop ND-1000 (Thermo Fisher Scientific, Massachusetts, The United States). Subsequently, the purified RNA was reverse transcribed into cDNA with HiScript III RT SuperMix (+ gDNA wiper) (#R323, Vazyme, Nanjing, China). Then, RT-qPCR was performed in Step One system with AceQ^®^ universal SYBR qPCR Master Mix (Vazyme). The gene expressions were calculated and normalized to the GAPDH gene. All of the primers were synthesized by Sangon Biotech (Shanghai, China) Co., Ltd.

### 2.11 Western blot

Cells were lysed by RIPA buffer and centrifuged (12,000 g × 30 min) to collect total protein in the supernatant, which were mixed with 5× SDS loading buffer (P0015, Beyotime, Shanghai, China) and heated to 100°C (10 min) for denaturation. After SDS-PAGE and transfer to the polyvinylidene difluoride (PVDF) membrane, the membranes were blocked by 5% skim milk solution and incubated with the corresponding primary antibodies at 4°C for a night. After three times wash, the membranes were incubated with secondary antibody and then washed for another three times, followed by chemiluminescent exposure of the blot with NcmECL Ultra (P10100, NCM Biotech, Suzhou, China). The primary antibodies were as follows: Anti-CD44 antibodies (15675-1-AP, Proteintech, Chicago, The United States), Anti-CD133 antibodies (18470-1-AP, Proteintech), Anti-SOX2 antibodies (#3579, Cell Signaling Technology, Massachusetts, The United States), Anti-C-MYC antibodies (#5605, Cell Signaling Technology), Anti-GAPDH antibodies (GB11002, Servicebio, Wuhan, China).

### 2.12 RNA sequencing

The total RNA was extracted from Lewis cells, Lewis cells cultured in 2% serum medium, and RT-MP-treated stem-like Lewis cells. RNA sequencing was performed by Wuhan Bioacme Biological Technology Co., Ltd.

### 2.13 Animal experiments

Male C57BL/6 J mice were bought from Liaoning Changsheng Biotechnology Co., Ltd. and maintained in the specific pathogen-free barrier facility in the Animal Center. To establish subcutaneous tumor bearing model, LLC-LUC cells cultured in 2% or 10% serum (1 × 10^6^ cells suspended in 100 μL PBS) were subcutaneously injected into the right flank of mice and administered intrapleural injections of 5 mg/kg DDP three times every 2 days. Tumor volumes were measured at the 15th day according to the formula *V* = (*L* × *W* ^2^)/2. In order to establish MPE model, mice were anaesthetized by 1% pentobarbital sodium. LLC-LUC cells (3 × 10^5^ cells suspended in 50 μL PBS) were injected into the right pleural cavity through the tenth or eleventh intercostal space at the midaxillary line. Four days later, all mice were observed by bioluminescence imaging to ensure that the MPE models were successfully and uniformly established. After that, the mice were randomized to 3 groups (control, RT-MPs, and RT-MPs + GSH) and intrapleurally injected with 50 μL liquid (PBS, RT-MPs or GSH-incubated RT-MPs) under isoflurane anaesthesia 4 times every 2 days. In order to evaluate MPE conditions, 2 mice in each group were imaged on day 20 through the Bruker *In Vivo* MS FX PRO Imager (Karlsruhe, Germany). The survival time of the remaining mice were observed.

### 2.14 Bioluminescence imaging

In order to evaluate MPE conditions, 2 mice in each group were anaesthetized with 1% pentobarbital sodium and injected intraperitoneally with 150 mg/kg firefly luciferin (CAS: 103404-75-7, Thermo Life, Massachusetts, The United States). And 10 min later, they were imaged with the Bruker *In Vivo* MS FX PRO Imager to acquire luminescent images (3-min exposure time) and X-ray photographs (30-s exposure time).

### 2.15 Quantification and statistical analysis

The statistical analysis was performed with Prism software (GraphPad Prism 9.0 software). Mine survival curves were analysed with the log-rank (Mantel-Cox) test. Statistical significance was analysed using the unpaired two-tailed Student’s t-test or two-way ANOVA. Data are presented as the mean ± SEM. Significant differences are indicated by *p < 0.05, **p < 0.01, ***p < 0.001, and NS: not significant.

## 3 Results

### 3.1 Low-serum culture promotes a quiescent state of tumor cells

**FIGURE 1:**
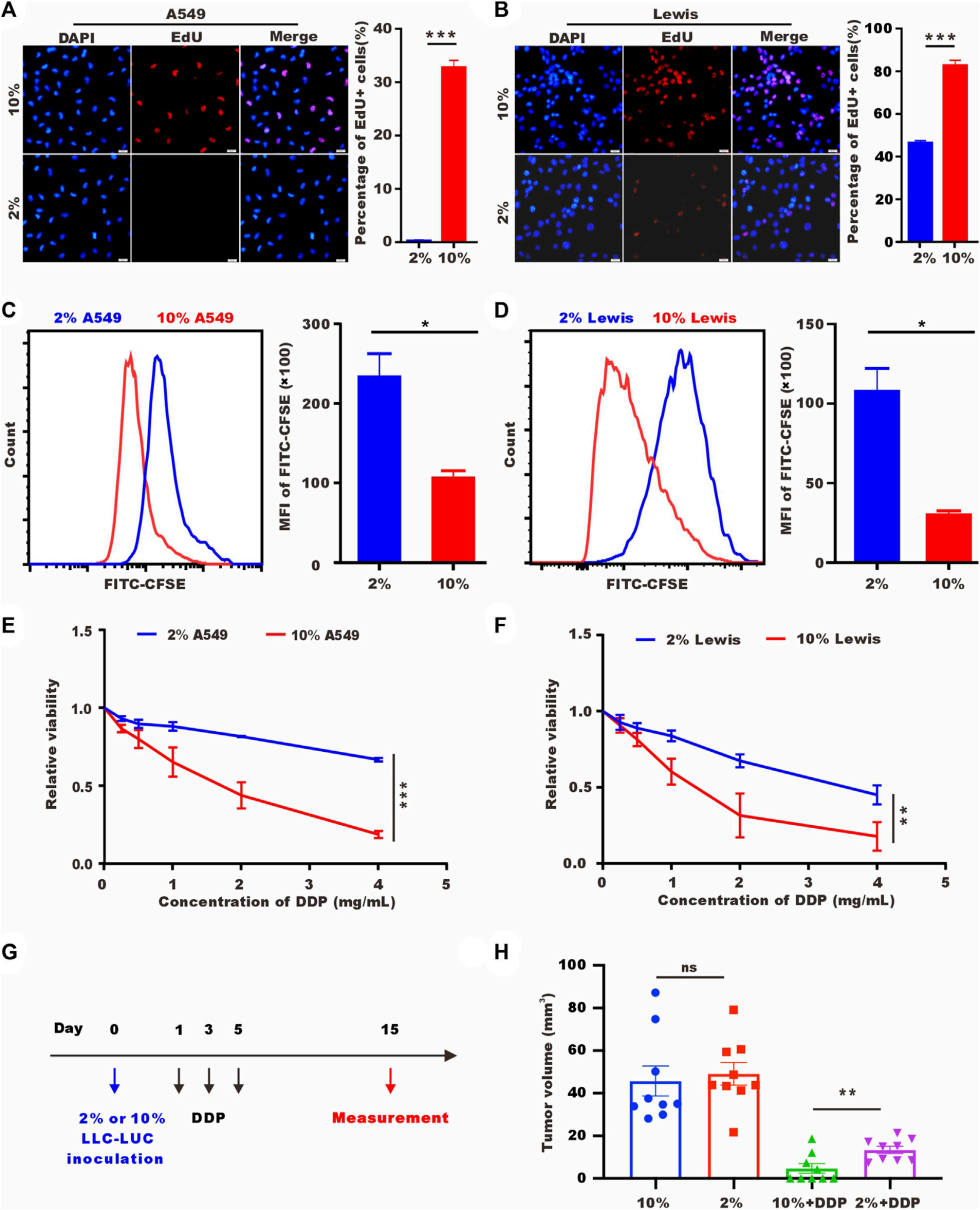
Low-serum culture induces tumor cell quiescence. **(A)** Representative images of EdU staining for A549 cells cultured in 10% serum or 2% serum for 72 h. Scale bar, 20 𝜇m. **(B)** Representative images of EdU staining for Lewis cells cultured in 10% serum or 2% serum for 72 h. Scale bar, 20 𝜇m. **(C,D)** CFSE assay of A549 cells and Lewis cells cultured in 10% serum or 2% serum culture for 72 h detected by flow cytometry. **(E,F)** A549 cells and Lewis cells were treated with different concentrations of DDP for 48 h, and cell viability was estimated by CCK-8 assays. **(G)** Treatment schedule of DDP for the mice with subcutaneous inoculation of LLC-LUC cells cultured in 10% serum or 2% serum. **(H)** Tumor volume measured in 15th day in tumor-bearing mice after DDP treatments (n = 8).

To verify the proliferation ability after 72 h of low-serum (2%) culture, we labelled A549 cells and Lewis cells with 5-ethynyl-20-deoxyuridine (EdU). As shown in Figures 1A, B, tumor cells cultured in low-serum medium showed significantly fewer EdU-positive cells. Staining for the proliferation marker Ki-67 provided the same results (Supplementary Figures S1A, B). We performed flow cytometry to detect carboxyfluorescein succinimidyl ester (CFSE), which decreased in fluorescence intensity as the cells divided, and discovered a higher mean value of CFSE in the low-serum-cultured A549 cells and Lewis cells (Figures 1C, D). These results indicated that A549 cells and Lewis cells underwent a rapid decrease in proliferation and entered a quiescence state under low-serum conditions. SLTCs were resistant to chemotherapy, and we examined the sensitivity of tumor cells cultured in low-serum medium to cisplatin (DDP) by using CCK-8 to test cell viability. As shown in Figures 1E, F, low-serum (2%)-cultured A549 cells and Lewis cells were more resistant to DDP. And we also proved this resistant effect of low-serum (2%)-cultured LLC-LUC cells *in vivo* (Figures 1G, H).

### 3.2 Low-serum culture induces high expression of stem cell-related genes

**FIGURE 2:**
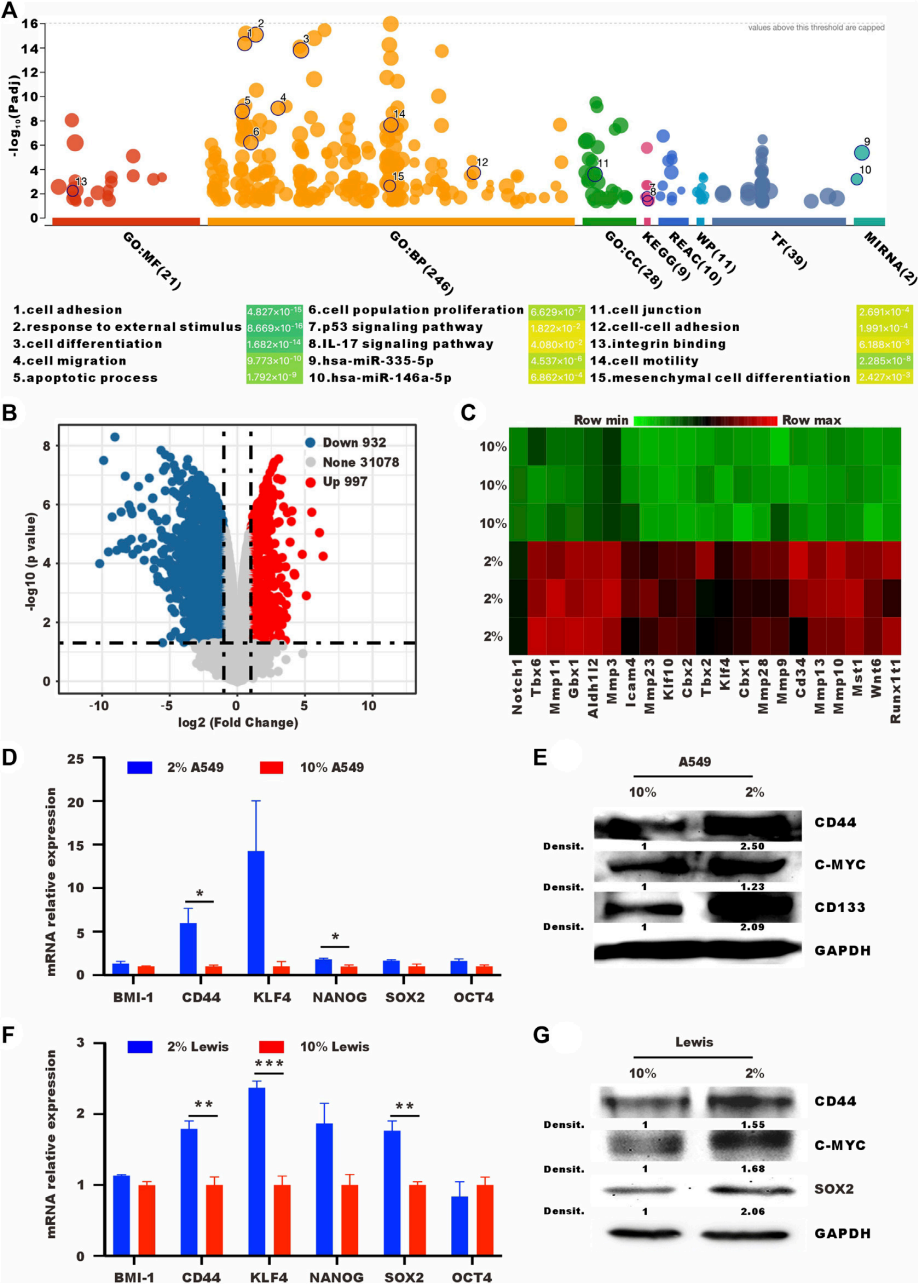
Tumor cells cultured in low serum have high expression levels of stem cell-related genes. **(A)** Bubble map of differentially enriched pathways. **(B)** Volcano plot showing the upregulated, downregulated or insignificantly differentially expressed genes in Lewis cells cultured in 10% or 2% serum medium for 72 h. **(C)** Heat map of differentially expressed stem cell-related genes in Lewis cells cultured in 10% or 2% serum medium for 72 h **(D)** RT-qPCR and **(E)** western blot analysis of the expression levels of stem cell-related in A549 cells cultured in 10% or 2% serum medium. **(F)** RT-qPCR and **(G)** western blot analysis of the expression levels of stem cell-related in Lewis cells cultured in 10% or 2% serum medium.

To understand the changes after low serum culturing at the transcriptional level, we performed RNA sequencing (RNA-seq) transcriptomic profiling under normal and low-serum conditions. Cell adhesion, response to external stimulus, cell differentiation, and cell migration were enriched in different databases. Low-serum culture induced the enrichment of stemness-related pathways, such as p53 signaling pathway and mesenchymal cell diffrentiation pathway (Pastushenko and Blanpain, 2019; Boutelle and Attardi, 2021) (Figure 2A). Lewis cells cultured in low serum had higher expression levels of stem cell-related genes, such as Notch1, Klf4, and Wnt6 (Figures 2B, C). We also performed RT-qPCR and western blot to analyse other stem-related markers, such as BMI-1, CD44, NANOG, SOX2, and OCT4 (Shi and Jin, 2010; Jia et al., 2019; Zhang et al., 2019). As shown in Figures 2D–G, 2% serum culture resulted in higher mRNA and protein levels of stem cell-related genes. In summary, 2% serum culture-induced A549 and Lewis cells showed vital features of SLTCs, including a propensity to enter proliferative quiescence and higher expression levels of stem cell-related genes.

### 3.3 RT-MPs effectively kill SLTCs

**FIGURE 3:**
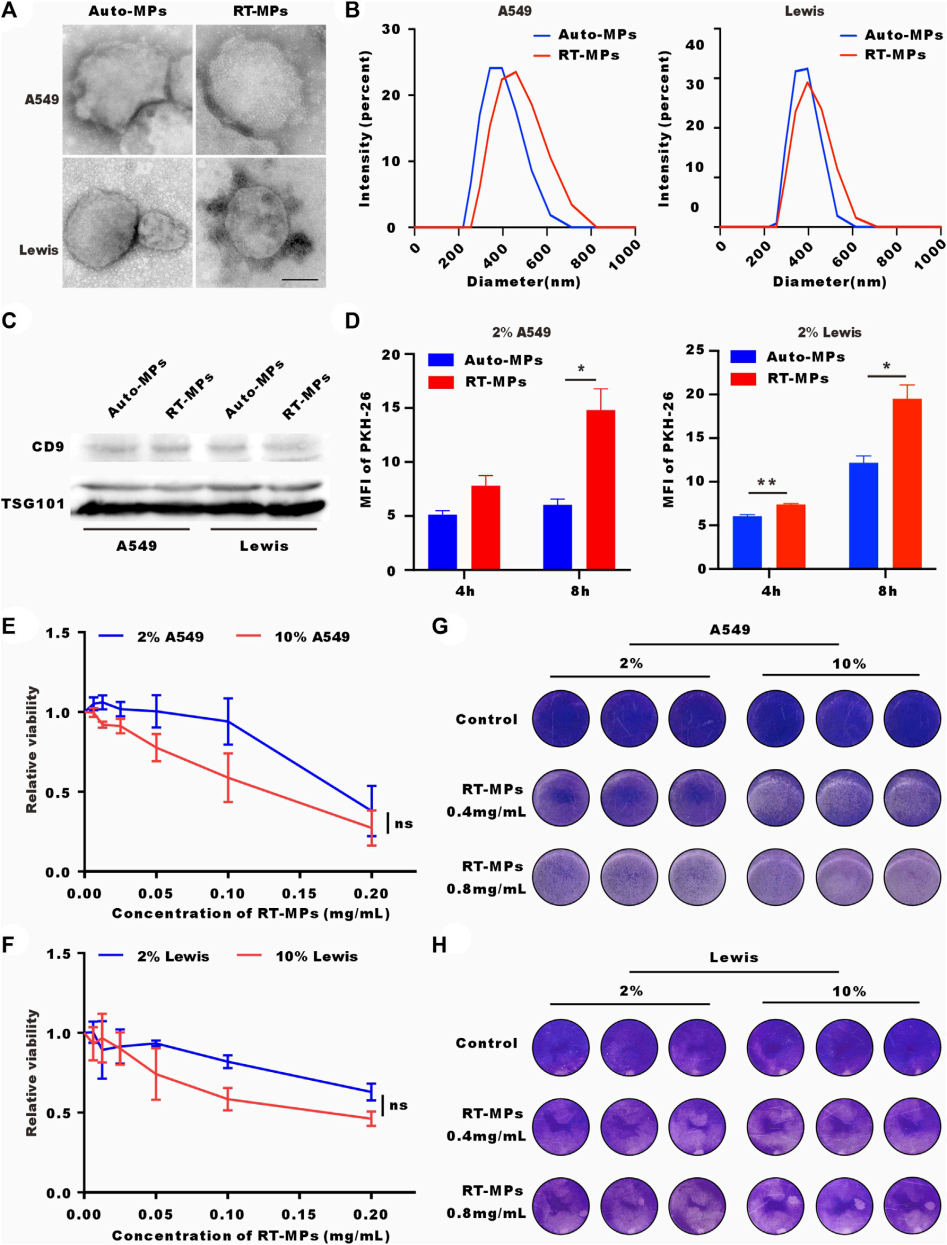
RT-MPs effectively kill SLTCs. **(A)** TEM images of RT-MPs. Scale bar, 200 nm. **(B)** Representative size and particle distribution plots of untreated A549 or Lewis-derived MPs and radiated A549 or Lewis-derived MPs. **(C)** Western blots of CD9 and TSG101 expression in untreated A549 or Lewis-derived MPs and irradiated A549 or Lewis-derived MPs. **(D)** Flow cytometric analysis of untreated A549 or Lewis-derived MPs and radiated A549 or Lewis-derived MP internalization by stem-like A549 and Lewis cells at multiple time points. **(E,F)** A549 and Lewis cells cultured in 10% or 2% serum medium were treated with different concentrations of RT-MPs for 48 h, and cell viability was estimated by CCK-8 assays. **(G,H)** Representative images of A549 cells and Lewis cells in the presence of different concentrations of RT-MPs (0.4 and 0.8 mg/mL) for 48 h.

Our group has reported that RT-MPs can induce radiation-induced bystander effects and cause tumor cell ferroptosis. Based on the finding that SLTCs are exposed to higher load of ROS and oxidative stress, we explored whether SLTCs are susceptible to RT-MPs, which can function by increasing the ROS level (Wang et al., 2018). We separated RT-MPs as previously reported and characterized them on the basis of morphology, size, and protein content. Transmission electron microscopy imaging showed that RT-MPs had a spherical morphology (Figure 3A). Nanoparticle tracking analysis revealed that untreated A549-and Lewis-derived RT-MPs had mean diameters of 383 nm and 376 nm and radiated A549-and Lewis-derived RT-MPs had mean diameters of 455 nm and 408 nm, respectively (Figure 3B). Western blot analysis revealed the presence of extracellular vesicle-associated proteins that are commonly used as extracellular vesicle markers, such as CD63, CD9 and tumor susceptibility gene 101 protein (TSG101) (Figure 3C).

We also investigated the uptake ability of SLTCs for RT-MPs by flow cytometry. Compared with untreated tumor cell-derived microparticles, stem-like A549 and Lewis cells internalized more RT-MPs (Figure 3D). We then explored the killing effect of RT-MPs toward SLTCs by using a CCK-8 kit assay and the colony formation assay. As shown in Figures 3E–H, RT-MPs could effectively kill both normal-serum-cultured and low-serum-cultured A549 and Lewis cells, and there was no significant difference in the killing effect under the two conditions.

### 3.4 SLTCs internalize fewer RT-MPs

**FIGURE 4:**
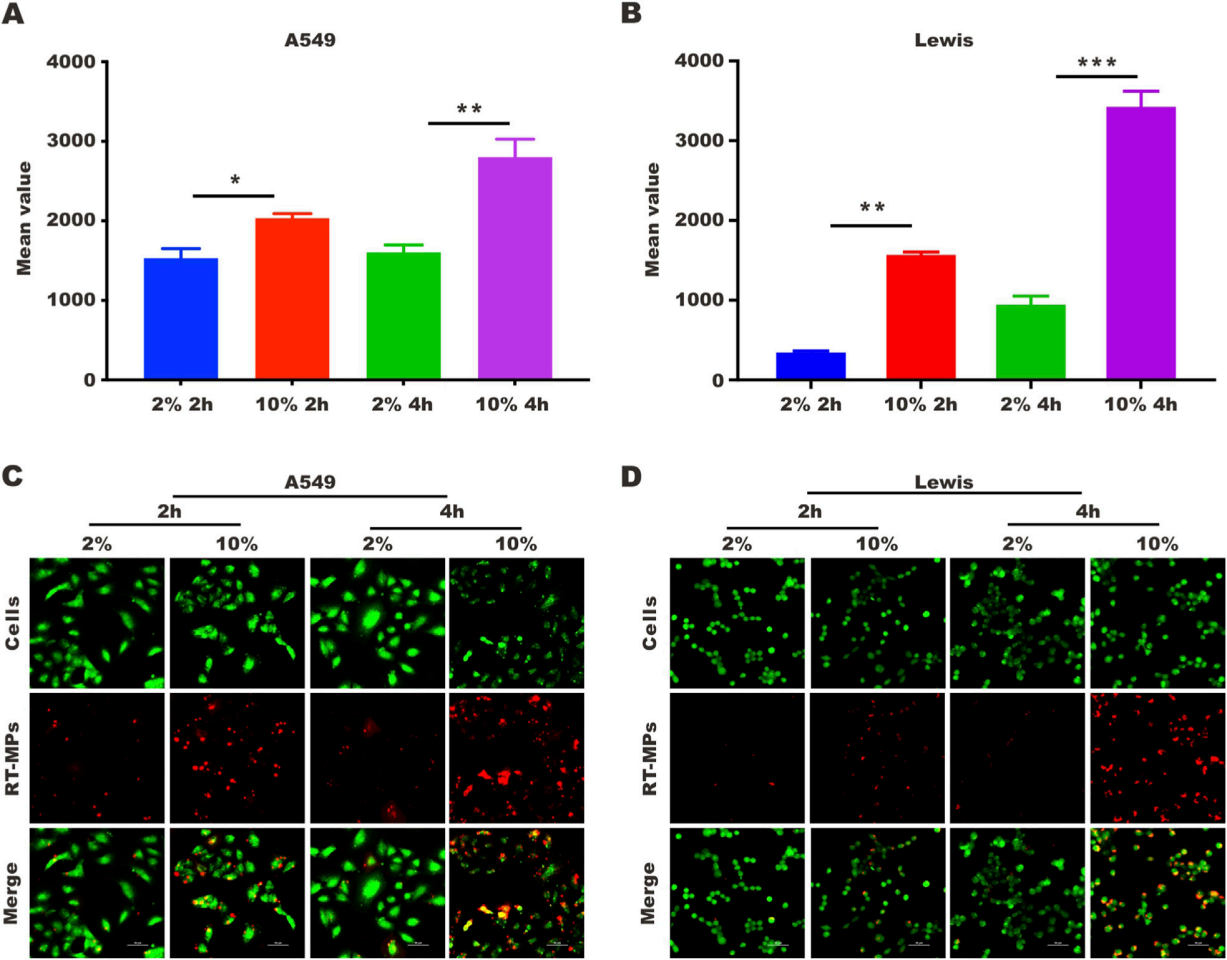
SLTCs internalize fewer RT-MPs. **(A,B)** Flow cytometric analysis of RT-MPs internalization by normal or stem-like A549 cells and normal or stem-like Lewis cells at multiple time points. **(C,D)** Representative images of tumor cells (green) phagocytosing red RT-MPs over time. A549 and Lewis cells were stained with CFSE, and RT-MPs were stained with the red fluorescent dye PKH26. Scale bar, 50 μm.

To explore the underlying mechanism by which RT-MPs kill SLTCs, we first examined the number of RT-MPs taken up by tumor cells under normal- and low-serum conditions by flow cytometry and confocal imaging. We labelled RT-MPs with PKH-26, added them to cells under two different conditions, and collected them after two and 4 hours. As shown in Figures 4A, B, SLTCs captured much fewer RT-MPs than standard-serum-cultured A549 or Lewis cells. It was clear that fewer red signals merged with the green signals in the low-serum culture group, indicating that fewer RT-MPs were internalized by the SLTCs (Figures 4C, D).

### 3.5 RT-MPs kill SLTCs by further increasing ROS levels

**FIGURE 5:**
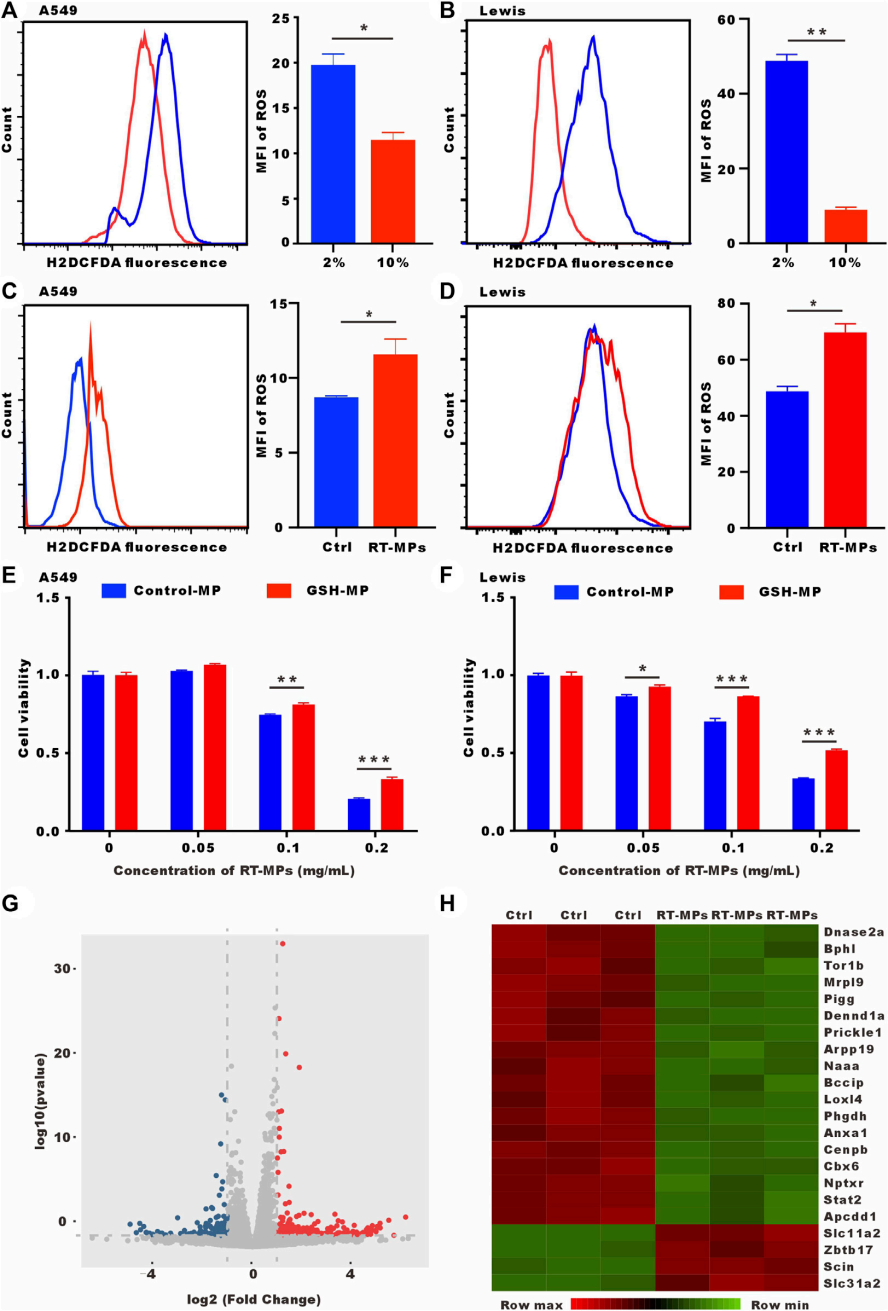
RT-MPs kill SLTCs by further increasing ROS levels. **(A,B)** Cytosolic ROS were assessed by flow cytometry using H2DCFDA. **(C,D)** ROS levels were assessed by flow cytometry in RT-MPs-treated stem-like A549 and Lewis cells. **(E,F)** Results of the CCK-8 assay for stem-like A549 and Lewis cells treated with RT-MPs and GSH-incubated RT-MPs. **(G)** Volcano plot showing significantly or insignificantly differentially expressed genes in stem-like Lewis cells untreated or treated with RT-MPs for 48 h. **(H)** Heat map of differentially expressed genes in stem-like Lewis cells untreated or treated with RT-MPs for 48 h.

It has been reported that SLTCs have high ROS levels. We verified that low-serum-cultured A549 and Lewis cells had significantly higher ROS levels (Figures 5A, B). RT-MPs further increased ROS levels under low-serum conditions (Figures 5C, D). Our group has discovered that RT-MPs contain ROS. To determine whether RT-MPs killed SLTCs through ROS, we incubated L-glutathione (GSH) with RT-MPs for 12 h following by washing three times with PBS, which significantly decreased the ROS levels in RT-MPs (Supplementary Figures S2A, B). GSH-incubated RT-MPs maintained a similar mean diameter of spherical structure with RT-MPs (Supplementary Figures S3A, B). The cell viability test showed that GSH-incubated RT-MPs could partially rescue the death caused by RT-MPs in stem-like A549 and Lewis cells (Figures 5E, F and Supplementary Figure S2C). To further elucidate the mechanism underlying the RT-MPs killing effect on SLTCs, we performed RNA-seq on stem-like Lewis cells and RT-MP-treated stem-like Lewis cells (Figures 5G, H). RT-MPs significantly upregulated the expression of ferroptosis-related gene Slc11a2 and DNA damage-related gene Zbtb11 in SLTCs (Seoane et al., 2002; Chen et al., 2021), which may provide insight into the further mechanisms underlying the cytotoxic effects of RT-MPs on SLTCs.

### 3.6 *In vivo* killing effect of RT-MPs on SLTCs

**FIGURE 6:**
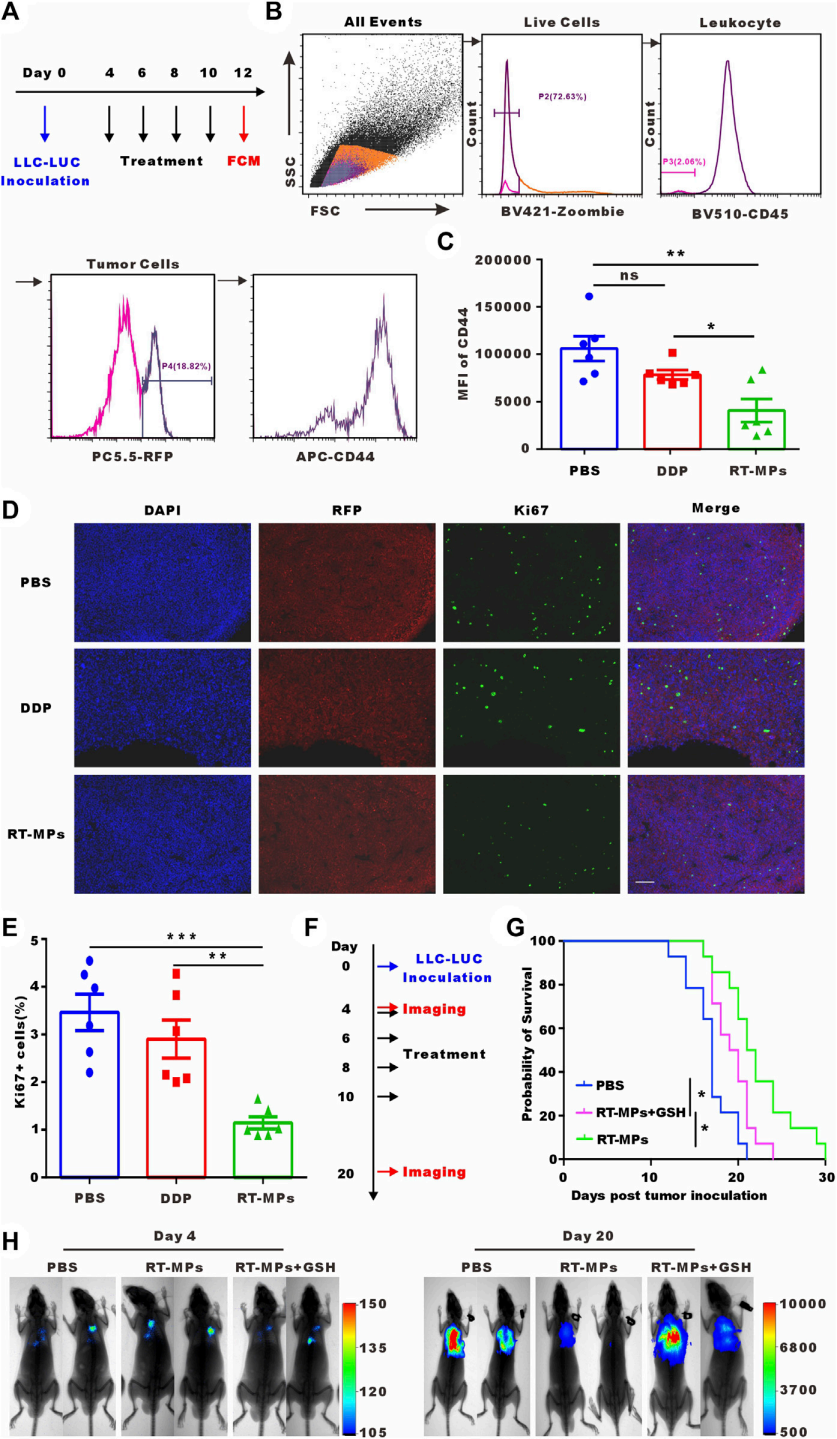
*In vivo* killing effect of RT-MPs on SLTCs. **(A)** Experimental outline for animal experiment. **(B)** Representative flow cytometry peak diagram of CD44 expression in tumor cells in MPE mice. **(C)** Quantification of expression levels. **(D)** Representative images of Ki67 immunofluorescence staining in pleural tumor nodules in the different groups. Scale bar, 100 µm. **(E)** Quantification of the proportion of Ki67-positive cells. **(F)** Experimental outline for animal experiment. **(G)** Kaplan-Meier survival plot of MPE mice in the corresponding treatment groups (n = 13–15 per group). **(H)** Representative *in vivo* bioluminescence images of the growth of mouse MPE under different treatment conditions.

We next investigated the killing effect of RT-MPs on SLTCs *in vivo*. We established a malignant pleural effusion (MPE) mouse model using Lewis cells stably transfected with red fluorescent protein (Lewis-RFP) and administered intrapleural injections of 3 mg/kg DDP and 0.1 mg/kg RT-MPs three times every 2 days. Three days after treatment was completed, cells were collected from the pleural lavage of MPE mice. We analysed the expression of the cancer stem cell marker CD44 in Lewis-RFP tumor cells (Figures 6A, B). The flow cytometry results showed that RT-MPs significantly decreased the percentage of CD44-positive tumor cells, while DDP did not (Figure 6C). We also measured Ki67 expression in pleural tumor cells in the three groups. As shown in Figures 6D, E, Ki67-positive cells decreased in mice treated with RT-MPs but not in mice treated with DDP. We further investigated the therapeutic effect of RT-MPs and the effect of GSH-incubated RT-MPs. As shown in Figures 6F, G, we observed increased survival upon treatment with RT-MPs, which was superior to that of the control and GSH-incubated RT-MPs treatments. GSH-incubated RT-MPs also prolonged the survival of MPE mice compared with the PBS group. *In vivo* imaging showed the tumor burden on day four and day 20 after inoculation with LLC-LUC cells, which was consistent with the survival curve (Figure 6H).

## 4 Discussion

Metastasis and recurrence are the main challenges in modern tumor treatment, and they arise from residual disseminated tumor cells that evade therapies and enter a dormant state (Miao et al., 2019; Pantel and Alix-Panabieres, 2019; Senft and Jeremias, 2019; Hen and Barkan, 2020). Understanding the mechanisms by which tumor enter a dormant state and finding ways to inhibit or kill SLTCs are very important to prevent metastasis and recurrence. Therapies targeting proliferating tumor cells have gained success. However, preclinical studies have shown that “awakening” dormant cells and then killing them with antiproliferation drugs rapidly fuel tumor recurrence (Essers and Trumpp, 2010; Bragado et al., 2013). The residual SLTCs are genetically heterogeneous, and awakening them would expand genetics and probably epigenetics and cause therapy resistance (Schmidt-Kittler et al., 2003; Stoecklein et al., 2008). Killing SLTCs in the dormant state would be a novel strategy to inhibit metastasis and recurrence.

RT-MPs are extravesicles that secreted by radiated tumor cells. Various treatments, including radiotherapy, chemotherapy, hypoxia, and ultraviolet radiation, can stimulate the release of microparticles (MPs) from tumor cells (Zhang et al., 2018; Chen et al., 2020). While most previously reported tumor-derived MPs have shown a prometastatic potential, our study suggests that RT-MPs exhibit unique antitumor effects. In our previous study, we demonstrated that RT-MPs killed tumor cells by ferroptosis and regulated the suppressive immune environment, but the specific killing mechanism is unknown. In this study, we found that RT-MPs could effectively kill SLTCs and that ROS played a significant role in this process. Other possible mechanisms require further exploration. SLTCs were found to have higher ROS levels and the induction of further accumulation of ROS may lead to the preferential killing of SLTCs. Determining ways to further increase ROS in SLTCs is a strategy for clearing SLTCs.

Local treatment with RT-MPs has been explored and has been shown to be very safe in thoracic injection, but this kind of injection still limits its clinical application. We further found that intravenous injection of RT-MPs was safe in a mouse model, but strategies to target and kill tumors after intravenous injection are being examined in further research.

## Data Availability

The datasets presented in this study can be found in online repositories. The names of the repositories and accession numbers can be found below: NCBI, GSE232958 and GSE233577.
