# Correction: Radiated tumor cell-derived microparticles effectively kill stem-like tumor cells by increasing reactive oxygen species

**DOI:** 10.3389/fbioe.2026.1795435

**Published:** 2026-03-03

**Authors:** Yan Hu, Chao Wan, Xiao Yang, Yu Tian, Suke Deng, Dandan An, Yijun Wang, Jiacheng Wang, Zhiyun Liao, Jingshu Meng, You Qin, Yajie Sun, Kunyu Yang

**Affiliations:** Cancer Center, Union Hospital, Tongji Medical College, Huazhong University of Science and Technology, Wuhan, China

**Keywords:** radiated tumor cells-derived microparticles, stem-like tumor cells, chemotherapy resistance, quiescence, reactive oxygen species

There was a mistake in Figure 4D as published. The Merge image corresponding to the 2-hour time point under 10% serum concentration in A549 cells was inadvertently duplicated. The corrected Figure 4D appears below.

**FIGURE 4:**
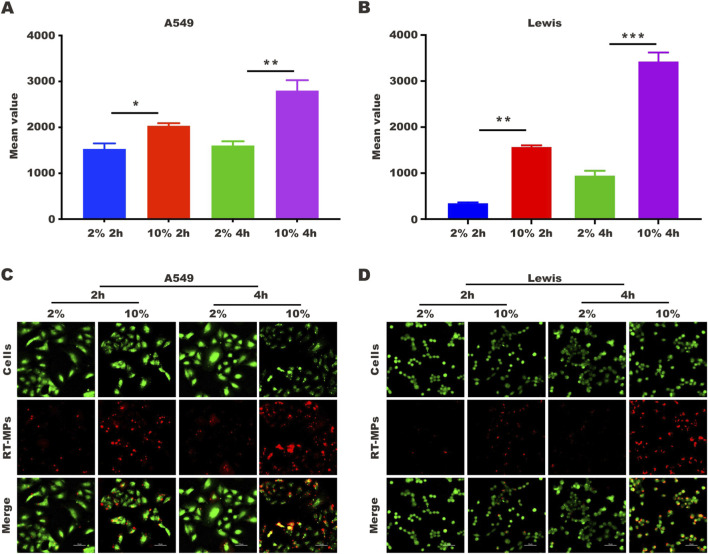
SLTCs internalize fewer RT-MPs. **(A,B)** Flow cytometric analysis of RT-MPs internalization by normal or stem-like A549 cells and normal or stem-like Lewis cells at multiple time points. **(C,D)** Representative images of tumor cells (green) phagocytosing red RT-MPs over time. A549 and Lewis cells were stained with CFSE, and RT-MPs were stained with the red fluorescent dye PKH26. Scale bar, 50 µm.

The original article has been updated.

