# Supplementary material for: Radiated tumor cell-derived microparticles effectively kill stem-like tumor cells by increasing reactive oxygen species
Source: Front Bioeng Biotechnol. 2023 Jun 5;11:1156951. doi: 10.3389/fbioe.2023.1156951 (PMC10277801; doi:10.3389/fbioe.2023.1156951)
Supplement: Supplementary file 1 [file DataSheet1.docx]

Supplementary Material

Radiated Tumor Cell-Derived Microparticles Effectively Kill Stem-like Tumor Cells by Increasing Reactive Oxygen Species

Yan Hu ^1†^, Chao Wan^1†^, Xiao Yang^1†^, Yu Tian^1^, Suke Deng^1^, Dandan An^1^, Yijun Wang^1^, Jiacheng Wang^1^, Zhiyun Liao^1^, Jingshu Meng^1^, You Qin^1^, Yajie Sun^1*^, Kunyu Yang^1*^

*** Correspondence:** Kunyu Yang: yangky71@aliyun.com; Yajie Sun: sunyajie@hust.edu.cn

## Supplementary Figures

##
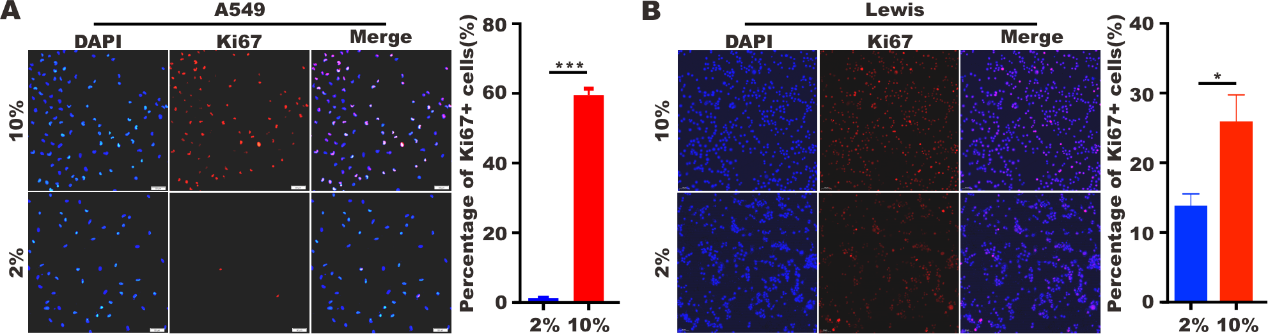


Supplementary Figure 1. (A-B) Representative images of Ki67 staining for A549 cells and Lewis cells cultured in 10% serum or 2% serum for 72 h. Scale bar, 50 𝜇m.


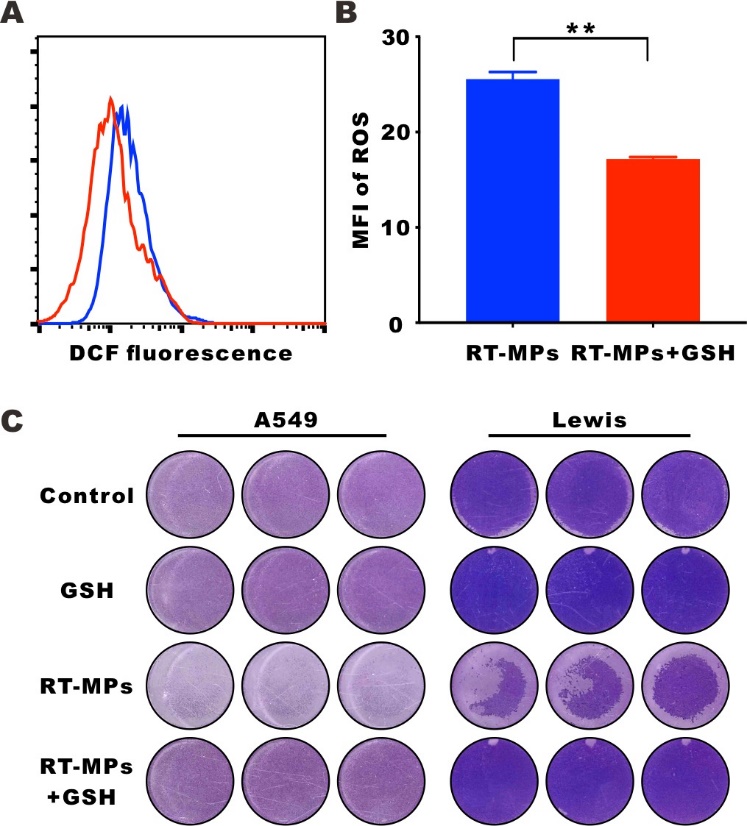


**Supplementary Figure 2. (A-B)** Cytosolic ROS in microparticles assessed by flow cytometry using H2DCFDA. **(C)** Representative images of A549 and Lewis cells in the presence of different treatments for 48 h.


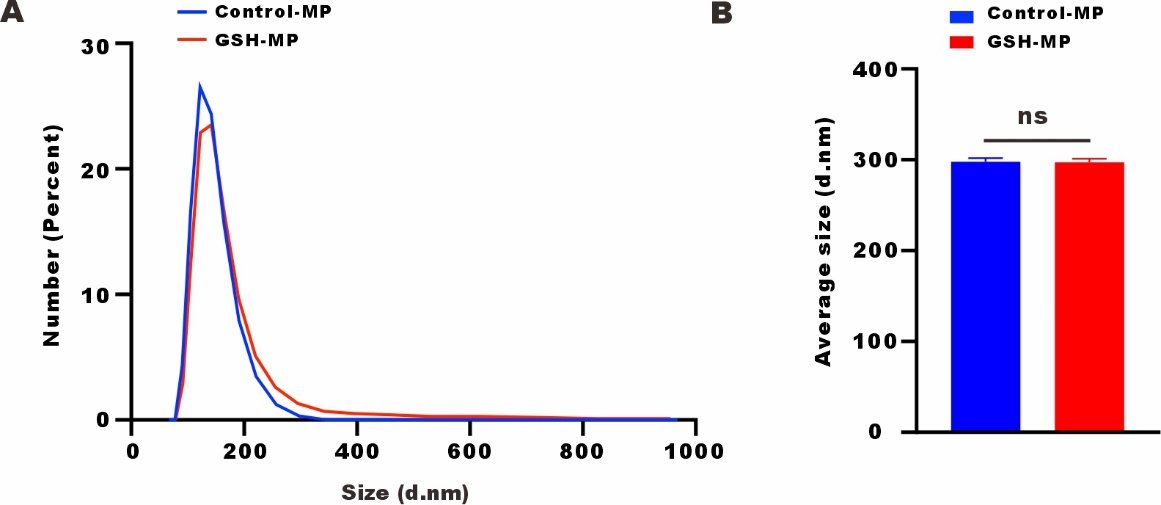


**Supplementary Figure 3. (A-B)** Representative size and particle distribution plots of RT-MPs and GSH-incubated RT-MPs.
